# Perceived discrimination and quality of life of the adult population from three cities in the Peruvian highlands

**DOI:** 10.17843/rpmesp.2024.414.13615

**Published:** 2024-10-22

**Authors:** Ybeth Luna-Solis, Horacio Vargas-Murga, Alberto Perales Cabrera, Ysela Agüero-Palacios, Carlos H. Contreras-Pizarro, Sara de la Torre Castillo

**Affiliations:** 1 Honorio Delgado-Hideyo Noguchi National Institute of Mental Health, Lima, Peru. Honorio Delgado-Hideyo Noguchi National Institute of Mental Health Lima Perú; 2 Universidad Nacional Mayor de San Marcos, Lima, Peru. Universidad Nacional Mayor de San Marcos Universidad Nacional Mayor de San Marcos Lima Peru; 3 Universidad Peruana Cayetano Heredia, Lima, Peru. Universidad Peruana Cayetano Heredia Universidad Peruana Cayetano Heredia Lima Peru; 4 National Academy of Medicine, Lima, Peru. National Academy of Medicine Lima Peru; 5 San Fernando Scientific Society, Lima, Peru. San Fernando Scientific Society Lima Peru

**Keywords:** Perceived Discrimination, Quality of Life, Population, Cities, Peru

## Abstract

In order to determine the relationship between perceived discrimination and quality of life, we carried out a cross-sectional analytical study of secondary sources. Data from three cities in the Peruvian highlands were analyzed. The sample of 3889 adults was grouped into participants with mental disorders (n=1499) and without mental disorders (n=2390). We used statistical tests of independence and independent group comparison for complex samples. The lowest quality of life was found in the group of participants with mental disorders who perceived themselves discriminated in the last year by sex (p<0.001), age (p<0.001), weight (p<0.001), dress (p<0.001), economic or social status (p<0.001), educational level (p<0.001), religion (p=0.008), friendships (p=0.005) and height (p=0.008). In conclusion, people with a mental disorder in the cities of Ayacucho, Cajamarca and Huaraz who perceived discrimination during the last year had lower quality of life averages than those without a mental disorder.

## INTRODUCTION

Perceived discrimination is the perception of a negative attitude or differential treatment due to certain particular characteristics of people according to sex, age, social status, among others, and is related to negative health outcomes ^1^. Likewise, quality of life is a construct of psychological well-being, considered equivalent to the state of health and includes external and individual components ^2^.

The relationship between perceived discrimination and quality of life has been studied in patients with chronic diseases such as cancer ^3^^)^ or diabetes ^4^, and significant relationships have been found when the perceived discrimination was based on race or ethnicity ^3^. Thus, a significant relationship has been found between greater stigma and worse quality of life in people with a mental disorder ^5^.

In Peru, disability faced by people with mental disorders is important, occupying the second place in the study of disease burden in 2019 ^6^, therefore studies on discrimination in the highlands become relevant because they are scarce and focus on people with mental disorders who have perceived discrimination for reasons that are not usually studied, as well as their relationship with quality of life ^7^.

Based on the above, this study aimed to determine the relationship between perceived discrimination and quality of life in the adult population with a mental disorder in Ayacucho, Cajamarca and Huaraz, three cities in the Peruvian highlands.

**KEY MESSAGES**

Motivation for the study. Discrimination as a negative social determinant for health entails limitations in several areas, even affecting people’s quality of life. Its research is mainly aimed at the racial context. Studies on other types of discrimination are scarce in the scientific literature.

Main findings. People with mental disorders perceived discrimination due to different sociodemographic conditions and had lower quality of life scores.

Implications. Discrimination as exclusionary behavior is facilitated and becomes more complex in people with some vulnerability.

## THE STUDY

### Study design

This is a secondary-data, cross-sectional, analytical research from the 2017 Epidemiological Study of Mental Health in Ayacucho, Cajamarca and Huaraz of the National Institute of Mental Health “Honorio Delgado Hideyo Noguchi” (INSM “HD-HN”) ^7^.

### Population and selection criteria

The primary study consisted of adults aged 18 years and older, usual residents in the cities of Ayacucho, Cajamarca and Huaraz. Direct and indirect interviews were conducted (in case of communication problems). Those who did not wish to participate were excluded.

### Sample size and sampling

The primary study considered an infinite population, obtaining a final sample of 3889 adults ^7^. The sampling was probabilistic, complex and tri-stage (clusters of dwellings, dwellings and persons), the unit of analysis being the person who met the selection criteria of the primary study ^7^. Each city in the study constituted a stratum, and in each dwelling more than one person could be selected from different units of analysis (adult, elderly, female unit and adolescent) ^7^.

### Variables

*Person with a mental disorder*

This variable was elaborated from other variables that are part of the MINI survey (International Neuropsychiatric Survey) Spanish version 5.0.0, linguistically adapted for the epidemiological studies of the INSM “HD-HN” ^7^. The mental disorders considered for this study are detailed in the supplementary material (S1).

*Perceived discrimination*

Attitude of devaluation towards others, ranging from rejection to violent behavior, without recognizing equal rights among people ^7^. It is measured on the basis of the Discrimination Questionnaire ^8^. This variable was incorporated into the epidemiological questionnaires of the INSM “HD-HN” from the epidemiological study of mental health in the highlands in 2003 ^9^, and analyzed for its validity and reliability (Cronbach’s α=0.670) with the population of Iquitos, Pucallpa and Tarapoto ^10^. The questionnaire asks about the prevalence in life and in the last 12 months of feeling rejected or discriminated against for different characteristics ^7^. For this study, we considered only the last year: “In the last 12 months, how often have you been rejected or discriminated against because of your sex, age, weight, skin color, dress, economic or social status, educational level, religion, friends, place of birth, height? from which the prevalence in the last year was estimated”. This last question was answered on a Likert scale (never, rarely, occasionally, frequently, very frequently) and, for the purposes of this study, was categorized as a dichotomous variable yes and no, with those who answered “never” corresponding to the “no” category. The content of the questionnaire and adaptations can be found in the supplementary material (S3).

*Quality of Life*

The Mezzich Quality of Life index, created in Spanish, was adapted and validated in Metropolitan Lima, and has a Cronbach’s α reliability of 0.870. It evaluates 10 areas by means of a single question with a Likert scale response (ranging from 1=bad to 10=excellent) ^11^^,^^12^. The content of the questionnaire and adaptations can be found in the supplementary material (S4).

*Sociodemographic characteristics of the population*

Sex, age, educational level, religion and poverty level were included according to self-perceived coverage of basic needs with family income ^7^. The categories and adaptations for the study can be found in the supplementary material (S2).

### Statistical analysis

Data was processed using the complex samples module of the SPSS V.21.0 program; this analysis was performed with weighted data. Frequencies and percentages were used for qualitative variables, and median and interquartile range for quality of life. The prevalences of the different types of discrimination between the two subgroups were compared with chi-square tests converted to the F statistic, and to compare the dimensions of quality of life and to evaluate the differences between the medians of quality of life, according to discrimination in the last year, we applied the Mann Whitney U test. The significance level (p value) was set at <0.05.

### Ethical Aspects

Since this is a secondary study, it was exempted from review with Official Letter No. 43-2023-CIEI/INSM “HD-HN” by the Institutional Research Ethics Committee (CIEI) of the INSM “HD-HN”. Likewise, the primary study, from which the anonymized database was obtained, was approved with document N°002-2016-CIEI/INSM “HD-HN” by the CIEI of the same institution.

## FINDINGS

The total number of participants was 3889 adults, of whom 1499 reported having some mental disorder. Table 1 shows the sociodemographic characteristics of the adults in the cities of Ayacucho, Cajamarca and Huaraz.

**Table 1:** Sociodemographic characteristics of the adult population of the cities of Ayacucho, Cajamarca and Huaraz, 2017.

Characteristics of the population		Total sample		Mental disorder (n=1499)		No mental disorder (n=2390)	
		n	%	n	%	n	%
Sex							
	Male	1550	47.35	577	45.50	973	48.46
	Female	2339	52.65	922	54.50	1417	51.54
Age group							
	18 - 24	748	21.15	229	16.88	519	23.72
	25 - 44	1810	42.14	638	38.55	1172	44.30
	45 - 64	882	26.42	389	30.61	493	23.90
	65 or more	449	10.29	243	13.96	206	8.08
Level of education^a^							
	No education/Kindergarten / Preschool	201	5.06	110	7.05	91	3.86
	Primary school	606	14.78	259	17.37	347	13.23
	Secondary school/high school	1171	29.13	457	29.43	714	28.94
	Non-university higher education	594	14.52	205	12.97	389	15.45
	University/postgraduate	1316	36.51	468	33.17	848	38.52
Religion							
	Catholic	2866	73.45	1098	72.57	1768	73.98
	Evangelical	587	14.66	228	15.36	359	14.24
	Other religions	232	6.41	89	6.36	143	6.44
	None	204	5.48	84	5.72	120	5.33
Poverty level according to self-perceived coverage of basic needs with family income at the end of the year a							
	Extremely poor	55	1.21	27	1.21	28	1.20
	Basic poor	860	21.26	361	22.32	499	20.62
	Basic non-poor	2060	53.15	756	52.46	1304	53.57
	Non-poor	904	24.38	353	24.01	551	24.61
Cities							
	Ayacucho	1188	22.70	567	45.28	728	36.39
	Cajamarca	1295	39.73	456	31.17	950	41.42
	Huaraz	1406	37.57	476	23.55	712	22.18

a Missing values (missing information)

### Perceived discrimination according to type of discrimination

The proportion of discrimination in the last year in the group of people who reported a mental disorder compared to those who did not was significant, with the exception of discrimination based on religion (p=0.802) and friendships (p=0.151) (Table 2).

**Table 2:** Prevalence of annual perceived discrimination according to types of discrimination in the adult population that reported having some mental disorder in the cities of Ayacucho, Cajamarca and Huaraz, 2017.

Perceived Annual Discrimination by Type of Discrimination		Total sample		Mental disorder		No mental disorder		p-value ^a^
		n=3889	%	n=1499	%	n=2390	%	
Sex								
	Yes	79	2.0	42	2.8	37	1.6	0.028
	No	3809	98.0	1457	97.2	2352	98.4	
Age								
	Yes	127	3.5	75	5.2	52	2.5	0.001
	No	3762	96.5	1424	94.8	2338	97.5	
Weight								
	Yes	118	3.5	68	5.2	50	2.5	0.001
	No	3768	96.5	1431	94.8	2337	97.5	
Skin color								
	Yes	42	1.1	24	1.7	18	0.7	0.015
	No	3847	98.9	1475	98.3	2372	99.3	
Way of dressing								
	Yes	95	3.0	52	4.3	43	2.1	0.007
	No	3792	97.0	1447	95.7	2345	97.9	
Economic or social status								
	Yes	143	3.5	76	5.0	67	2.7	0.001
	No	3743	96.5	1423	95.0	2320	97.3	
Educational level								
	Yes	113	2.9	66	4.5	47	1.9	<0.001
	No	3764	97.1	1426	95.5	2338	98.1	
Religion								
	Yes	59	1.8	25	1.9	34	1.7	0.802
	No	3813	98.2	1465	98.1	2348	98.3	
Friendships								
	Yes	42	1.6	22	2.2	20	1.2	0.151
	No	3846	98.4	1476	97.8	2370	98.8	
Place of birth								
	Yes	48	1.3	29	2.2	19	0.7	<0.001
	No	3840	98.7	1470	97.8	2370	99.3	
Height								
	Yes	64	2.0	39	3.7	25	1.1	<0.001
	No	3824	98.0	1459	96.3	2365	98.9

a Independence test for complex samples (chi-square tests converted to the F-statistic).

### Quality of life

The median quality of life of the studied population was 7.9 (IQR: 7.1-8.5) (Table 3). 

Adults with some mental disorder obtained a median of 7.6 (IQR: 6.8-8.3), which is lower compared to those who did not present any mental disorder 8.0 (IQR: 7.3-8.7), this difference being significant (p<0.01) (Table 3).

**Table 3:** Quality of life in adult population, who declared having some mental disorder, from the cities of Ayacucho, Cajamarca and Huaraz, 2017.

Quality of life	Total sample			Mental disorder			No mental disorder			p-value ^a^
	Median	Q1	Q3	Median	Q1	Q3	Median	Q1	Q3	
Average total quality of life	7.9	7.1	8.5	7.6	6.8	8.3	8.0	7.3	8.7	<0.001
Physical well-being	8.0	6.0	9.0	7.0	6.0	8.0	8.0	7.0	9.0	<0.001
Emotional psychological well-being	8.0	7.0	9.0	8.0	6.0	8.0	8.0	7.0	9.0	<0.001
Self-care and independent functioning	8.0	8.0	9.0	8.0	7.0	9.0	9.0	8.0	10.0	<0.001
Occupational performance	8.0	8.0	9.0	8.0	7.0	9.0	8.0	8.0	9.0	<0.001
Interpersonal performance	8.0	7.0	9.0	8.0	7.0	9.0	8.0	7.0	9.0	<0.001
Social emotional support	8.0	7.0	9.0	8.0	6.0	8.0	8.0	7.0	9.0	<0.001
Community and service support	7.0	5.0	8,0	6,0	5.0	8.0	7.0	6.0	8.0	<0.001
Personal fulfillment	8.0	7.0	9.0	8.0	6.0	8.0	8.0	7.0	9.0	<0.001
Spiritual satisfaction	8.0	7.0	9.0	8.0	7.0	9.0	8.0	8.0	9.0	<0.001
Overall quality of life	8.0	7.0	9.0	8.0	7.0	9.0	8.0	8.0	9.0	<0.001

a Mann-Whitney U test, Q1: quartile 1, Q3: quartile 3.

### Perceived discrimination and quality of life

For the population with a mental disorder, the average quality of life was lower when related to perceived discrimination in the last year for all types of discrimination (p<0.001) except skin color (p=0.326) and place of birth (p=0.838) (Table 4). There was significant difference in the average quality of life of people without mental disorder who perceived discrimination in the last year, the lowest averages of quality of life were related to perceived discrimination by weight (p<0.001), educational level (p=0.003), way of dressing (p=0.011), friendships (p<0.001), height (p=0.013) and economic or social condition (p=0.034) (Table 4).

**Table 4:** Relationship between annual perceived discrimination and quality of life in adult population in the cities of Ayacucho, Cajamarca and Huaraz, 2017.

Perceived annual discrimination		Quality of life														
		Total sample					Mental disorder					No mental disorder				
		n	Median	Q1	Q3	p-value ^a^	n	Median	Q1	Q3	p-value ^a^	n	Median	Q1	Q3	p-value ^a^
Sex																
	No	3763	7.90	7.20	8.50	<0.001	1444	7.60	6.80	8.30	<0.001	2316	8.00	7.40	8.70	0.050
	Yes	79	7.10	6.30	7.80		42	6.70	5.70	7.20		37	7.50	6.90	8.30	
Age																
	No	3717	7.90	7.20	8.50	<0.001	1412	7.60	6.90	8.30	<0.001	2302	8.00	7.40	8.70	0.304
	Yes	126	7.30	6.40	8.10		74	6.70	5.80	7.90		52	7.80	6.80	8.50	
Weight																
	No	3722	7.90	7.20	8.60	<0.001	1418	7.60	6.80	8.30	<0.001	2301	8.00	7.40	8.70	<0.001
	Yes	118	7.20	6.70	8.00		68	7.10	6.10	7.70		50	7.20	7.00	8.00	
Skin color																
	No	3801	7.90	7.10	8.50	0.272	1462	7.60	6.80	8.30	0.326	2336	8.00	7.30	8.70	0.946
	Yes	42	7,50	6.70	8.40		24	6.90	6.70	8.00		18	8.20	6.80	8.40	
Way of dressing																
	No	3746	7.90	7.20	8.50	<0.001	1434	7.60	6.80	8.30	<0.001	2309	8.00	7.40	8.70	0.011
	Yes	95	7.10	6.30	7.90		52	6.70	6.10	7.60		43	7.40	6.80	8.10	
Economic or social status ^a^																
	No	3697	7.90	7.20	8.50	<0.001	1411	7.60	6.80	8.30	0.001	2284	8.00	7.40	8.70	0.034
	Yes	143	7.30	6.40	8.20		75	7.00	6.40	7.90		67	7.90	7.10	8.40	
Educational level																
	No	3718	7.90	7.20	8.50	<0.001	1413	7.60	6.80	8.30	<0.001	2302	8.00	7.40	8.70	0.003
	Yes	113	7.20	6.40	8.00		66	7.10	5.80	8.00		47	7.30	6.80	8.20	
Religion																
	No	3766	7.90	7.10	8.50	0.248	1452	7.60	6.80	8.30	0.008	2312	8.00	7.30	8.70	0.413
	Yes	59	7.60	7.00	8.60		25	6.80	5.70	7.60		34	8.00	7.40	8.90	
Friendships																
	No	3800	7.90	7.10	8.50	<0.001	1463	7.60	6.80	8.30	0.005	2334	8.00	7.40	8.70	<0.001
	Yes	42	6.90	6.00	7.40		22	6.30	5.70	7.30		20	7.40	6.90	7.50	
Place of birth																
	No	3794	7.90	7.10	8.50	0.389	1457	7.60	6.80	8.30	0.838	2334	8.00	7.30	8.70	0.831
	Yes	48	7.90	6.70	8.50		29	7.50	6.40	8.50		19	8.00	6.80	8.70	
Height																
	No	3778	7.90	7.20	8.50	<0.001	1446	7.60	6.80	8.30	0.008	2329	8.00	7.40	8.70	0.013
	Yes	64	7.10	6.30	7.70		39	7.00	5.80	7.50		25	7.40	7.00	8.00	

a Mann-Whitney U test.

## DISCUSSION

Discrimination in the last year in people with a mental disorder was higher than in people without a mental disorder, a result similar to a national survey in Peru, where a higher perception of discrimination was found in people with intellectual or mental disabilities, among others ^13^. The study by Alonso *et al*. ^14^, in people with mental disorders, reported that 18% (n=1851) experienced discrimination, even more than those with chronic physical health problems.

The types of discrimination most frequently reported by adults with a mental disorder were socioeconomic or social status, age, weight and educational level. This coincides with the study by Amirova *et al*. ^15^, who found, in England, that discrimination based on economic status was the most frequent, and with a Chilean national survey, where discrimination based on socioeconomic status (21%) and age (17%) were the most prevalent ^16^. Amirova *et al*. ^15^^)^ also found that, in the United States, discrimination based on sex was the most frequent, mainly among women. Among our results, sex discrimination also stands out. In this regard, we should remember that women in various parts of the world are more exposed to sexism, which has a negative impact on their quality of life ^17^, so our findings may reflect part of the cultural idiosyncrasies of the Peruvian highland regions.

Significant differences were found in the quality of life between the two subsamples, with adults with mental disorders having lower median values, mainly in the dimensions of quality of life related to community support and physical well-being. These results are compatible with the study by Cedillo *et al*. ^18^, who found that the increase in the limitations in the functioning of the person with some type of disability decreased their quality of life. Likewise, in the study by Alonso *et al*. ^14^, lower quality of life in people with mental disorders was associated with perceived stigma.

Although it is difficult to make comparisons between perceived discrimination and quality of life in the population with a mental disorder due to the scarcity of studies, and the use of different scales, the study by Ng *et al*. ^19^, in 380 adult migrants in Hong Kong, reported a negative association between perceived discrimination and three dimensions of quality of life (physical, psychological and environmental health) of the four evaluated with the “WHOQOL-BREF” questionnaire. On the other hand, the different types of discrimination interact with each other and, mediated by the discriminatory experience, affect the quality of life of the people who perceive them ^17^. Although the approach of this study has not been part of our research, it is important to consider for future research.

Our study should be interpreted considering several limitations. First, being a secondary source study, the research question was answered by using existing data. Memory bias was another limitation (the questions draw on the interviewee’s recollections). There was also a possible measurement bias (the instruments were validated in Spanish, even though the study population included Quechua speakers with a different cultural context), and social desirability (due to the sensitive nature of the questions). It is worth mentioning that in the original study a pilot test was conducted to obtain valid and reliable information ^7^. There could be a problem of understanding discrimination based on friendship; on the one hand, it could refer to the friendships that a given person has, or that his or her friends discriminate against him or her for different reasons. Also, when the responses were grouped on a Likert scale of five to two categories, it was not possible to identify whether the higher the frequency of perceived discrimination, the lower the quality of life. Finally, it was not possible to associate perceived discrimination and quality of life according to the city, due to the absence of values in any of the three subgroups formed.

Beyond the above limitations, the study has several strengths, such as having a representative sample, and being one of the first to compare the relationship between perceived discrimination and quality of life in people with a mental disorder in cities in the Peruvian highlands, the study being meritorious because of the scarce information on how much perceived discrimination affects well-being.

In conclusion, people with mental disorders in the cities of Ayacucho, Cajamarca and Huaraz who perceived discrimination in the last year had lower quality of life averages than those without mental disorders. It is recommended to continue research on this social determinant that causes conflicts and negatively influences the consolidation of the country.
