# Supplementary material for: Perceived discrimination and quality of life of the adult population from three cities in the Peruvian highlands
Source: Rev Peru Med Exp Salud Publica. 2024 Oct 22;41(4):392–8. doi: 10.17843/rpmesp.2024.414.13615 (PMC11797587; doi:10.17843/rpmesp.2024.414.13615)
Supplement: Supplementary material. — Available in the electronic version of the RPMESP. [file rpmesp-41-04-13615-s001.docx]

| Variables | Estudio original | Adaptación para el artículo | Razón |
| --- | --- | --- | --- |
| Discriminación percibida (S3) |  |  |  |
| En los últimos 12 meses, ¿con qué frecuencia ha sido Usted rechazado(a) o discriminado(a) por su sexo, edad, peso, color de piel, forma de vestir, condición económica o social, nivel educativo, religión, amistades, lugar de nacimiento, talla? | Respuestas posibles:   - ninguna vez - rara vez, - ocasionalmente, - frecuentemente, - muy frecuentemente - no responde | Respuestas posibles:   - SÍ= muy frecuentemente, frecuentemente, ocasionalmente, rara vez - NO= ninguna vez | Aumentar la representatividad, debido a que había categorías con muy pocas respuestas e igualar a las categorías de la pregunta alguna vez en la vida. |
| Calidad de vida (S4) |  |  |  |
| Bienestar físico | Respuestas posibles  Desde 1=malo hasta 10=excelente | Sin modificaciones | |
| Bienestar psicológico |  |  |  |
| Autocuidado y funcionamiento independiente |  |  |  |
| Bienestar ocupacional |  |  |  |
| Funcionamiento interpersonal |  |  |  |
| Apoyo socio emocional |  |  |  |
| Apoyo comunitario |  |  |  |
| Plenitud personal |  |  |  |
| Satisfacción espiritual |  |  |  |
| Calidad de vida global |  |  |  |
| Características sociodemográficas (S2) |  |  | |
| Sexo | Respuestas posibles:   - Masculino - Femenino | Sin modificaciones | |
| Edad | - La unidad de análisis del adulto correspondió de 18 a más años de edad. | Se consideró a los mayores e iguales de 18 a más años en categorías de  18-24  25-44  45-64  65 a más | Al tener las demás variables en categorías se consideró pertinente la agrupación de edades y convertirla a una variable cualitativa como las demás. |
| Nivel de instrucción | Respuestas posibles:   - Sin nivel/nunca asistió a la escuela - Inicial/preescolar - Primaria - Secundaria - Bachillerato - Superior no universitario - Superior universitario - Posgrado | Respuestas posibles:   - Sin instrucción/Inicial/preescolar - Primaria - Secundaria/bachillerato - Superior no universitario - Superior universitario/posgrado | Aumentar la representatividad, debido a que había categorías con muy pocas respuestas. |
| Religión | Respuestas posibles:   - Católica - Evangélica - Testigo de Jehová - Adventista - Mormona - Ninguna - Otra | Respuestas posibles:   - Católica - Evangélica - Otra religión (testigo de Jehová, adventista, mormona y otras) - Ninguna | Aumentar la representatividad, debido a que había categorías con muy pocas respuestas. |
| Autopercepción de pobreza | Respuestas posibles:   - pobres extremos: no cubren las necesidades básicas de alimentación - pobres básicas: cubren las necesidades de alimentación, pero no otras como salud, educación, vivienda, etc. - no pobres básicas: cubren sus necesidades básicas, pero no otras como diversión, distracción, educación diferenciada - no pobres: cubren sus necesidades básicas y otras como pasatiempos, educación diferenciada, diversión, etc. | Sin modificaciones | |

| Unidad de análisis del presente estudio | Definición | Medición |
| --- | --- | --- |
| Persona con trastorno mental (S1) | Aquella que tenía indicadores de cualquiera de los siguientes problemas:   - Ansiedad - Depresión - Psicosis - Dependencia a cualquier sustancia incluida el alcohol - Problemas relacionados al interés por el sexo y relaciones sexuales (impotencia, eyaculación precoz, etc.) - Anorexia | A partir de la encuesta MINI Encuesta Neuropsiquiátrica Internacional (MINI) versión en español 5.0.0 adaptada lingüísticamente para los estudios epidemiológicos del INSM “HD-HN” **(7)** |
| Persona sin trastorno mental | Aquella que respondió no presentar indicadores de cualquiera de los trastornos mentales antes señalados. | No presentar indicadores de algún problema de salud mental en la encuesta MINI Encuesta Neuropsiquiátrica Internacional (MINI) versión en español 5.0.0 adaptada lingüísticamente para los estudios epidemiológicos del INSM “HD-HN” **(7)** |

**S3**

**CUESTIONARIO MODIFICADO (CONSIDERANDO SOLO LA PREGUNTA EN LOS ÚLTIMOS 12 MESES) SOBRE DISCRIMINACIÓN CONTENIDO EN EL MÓDULO DE SALUD INTEGRAL DEL ADULTO DEL ESTUDIO EPIDEMIOLÓGICO DE SALUD MENTAL DE AYACUCHO, CAJAMARCA Y HUARAZ-2017 DEL INSM “HD-HN”**

| 1. **¿en lOS últimoS 12 meses, con que frecuencia ha sido usted rechazado(a) o discriminado(a) por su.** | *ninguna vez* | *RARA VEZ* | *OCASIONALMENTE* | *FRECUENTEMEnTE* | *MUY FRECUENTE.* | *No RESPONDE* |
| --- | --- | --- | --- | --- | --- | --- |
| *a. Sexo?* | *1* | *2* | *3* | *4* | *5* | *6* |
| *b. Edad?* | *1* | *2* | *3* | *4* | *5* | *6* |
| *c. Peso?* | *1* | *2* | *3* | *4* | *5* | *6* |
| *d. Color de piel?* | *1* | *2* | *3* | *4* | *5* | *6* |
| *e. Forma de vestir?* | *1* | *2* | *3* | *4* | *5* | *6* |
| *f. Condición económica o social?* | *1* | *2* | *3* | *4* | *5* | *6* |
| *g. Nivel educativo?* | *1* | *2* | *3* | *4* | *5* | *6* |
| *h. Religión?* | *1* | *2* | *3* | *4* | *5* | *6* |
| *i. Amistades?* | *1* | *2* | *3* | *4* | *5* | *6* |
| *j. Lugar de nacimiento?* | *1* | *2* | *3* | *4* | *5* | *6* |
| *k. Talla?* | *1* | *2* | *3* | *4* | *5* | *6* |
| *l. Otro?(Especifique)*  *....................................................................* | *1* | *2* | *3* | *4* | *5* | *6* |

**S4**

**Calidad de vida**. Medido a partir de Índice de Calidad de Vida de Mezzich. El índice fue elaborado por Mezzich et al. **(10),** en idioma español, consta de 10 áreas: bienestar físico, bienestar psicológico, autocuidado y funcionamiento independiente, bienestar ocupacional, funcionamiento interpersonal, apoyo socio-emocional, apoyo comunitario, plenitud personal, satisfacción espiritual y la calidad de vida global. Este instrumento fue adaptado, validado e incluido como parte del Cuestionario de Salud Mental del Estudio Epidemiológico de Salud Mental en Lima Metropolitana **(11).** Consta de cuatro dimensiones y una confiabilidad α de Cronbach de 0.870 **(11).** El cuestionario está compuesto de 10 preguntas sobre la situación actual de las 10 áreas antes mencionadas, con respuesta en escala Likert (que va desde 1=malo hasta 10=excelente) **(11).**

**INDICE DE CALIDAD DE VIDA (ADAPTACIÓN MEZZICH Y COLABORADORES 2000) CONTENIDO EN EL MÓDULO DE SALUD INTEGRAL DEL ADULTO DEL ESTUDIO EPIDEMIOLÓGICO DE SALUD MENTAL DE AYACUCHO, CAJAMARCA Y HUARAZ-2017 DEL INSM “HD-HN”**

Con respecto a los siguientes aspectos de su vida, califique de 1 a 10 su situación actual, siendo 10 es excelente:

| 1. Bienestar Físico; es decir, sentirse con energía, sin dolores ni problemas físicos |  |
| --- | --- |
| 1. Bienestar Psicológico o emocional; es decir, sentirse bien y satisfecho consigo mismo. |  |
| 1. Autocuidado y funcionamiento independiente; es decir, cuidar bien de su persona, tomar sus propias decisiones. |  |
| 1. Funcionamiento ocupacional; es decir, ser capaz de realizar un trabajo remunerado, tareas escolares y/o domésticas. |  |
| 1. Funcionamiento interpersonal; es decir, ser capaz de responder y relacionarse bien con su familia, amigos y grupos. |  |
| 1. Apoyo social-emocional; es decir, poseer disponibilidad de personas en quien confiar y de personas que le proporcionen ayuda y apoyo emocional. |  |
| 1. Apoyo Comunitario; es decir, poseer un buen vecindario, disponer de apoyos financieros y de otros servicios. |  |
| 1. Plenitud personal; es decir, sentido de realización personal y de estar cumpliendo con sus metas más importantes. |  |
| 1. Satisfacción espiritual; es decir, haber desarrollado una actitud hacia la vida más allá de lo material y un estado de paz interior consigo mismo y con las demás personas. |  |
| 1. Calidad de vida global; es decir, sentirse satisfecho y feliz con su vida en general. |  |
| Total |  |
