# Supplementary material for: Perceived discrimination and quality of life of the adult population from three cities in the Peruvian highlands
Source: Rev Peru Med Exp Salud Publica. 2024 Oct 22;41(4):392–8. doi: 10.17843/rpmesp.2024.414.13615 (PMC11797587; doi:10.17843/rpmesp.2024.414.13615)
Supplement: Translation

# Translation: Discriminación percibida y calidad de vida en población adulta de tres ciudades de la sierra peruana

**Authors:** Ybeth Luna-Solis, Horacio Vargas-Murga, Alberto Perales Cabrera, Ysela Agüero-Palacios, Carlos H. Contreras-Pizarro, Sara de la Torre Castillo

**Affiliations:** 1 Instituto Nacional de Salud Mental Honorio Delgado-Hideyo Noguchi, Lima, Perú.; 2 Universidad Nacional Mayor de San Marcos, Lima, Perú.; 3 Universidad Peruana Cayetano Heredia, Lima, Perú.; 4 Academia Nacional de Medicina, Lima, Perú.; 5 Sociedad Científica de San Fernando, Lima, Perú.

**Keywords:** Discriminación Percibida, Calidad de Vida, Población, Ciudades, Perú

## Abstract

Con el objetivo de determinar la relación entre la discriminación percibida y la calidad de vida se realizó un estudio analítico transversal de fuente secundaria. Se analizaron los datos de tres ciudades de la sierra peruana. La muestra de 3889 adultos fue agrupada en adultos con trastornos mentales (n=1499) y sin trastornos mentales (n=2390). Se utilizaron pruebas estadísticas de independencia y de comparación de grupos independientes para muestras complejas. La menor calidad de vida se encontró en el grupo de personas con trastornos mentales que se percibió discriminado en el último año por sexo (p<0,001), por edad (p<0,001), peso (p< 0,001), forma de vestir (p<0,001), condición económica o social (p<0,001), nivel educativo (p<0,001), religión (p=0,008), amistades (p=0,005) y talla (p=0,008). En conclusión, las personas con algún trastorno mental de las ciudades de Ayacucho, Cajamarca y Huaraz que percibieron discriminación en el último año presentaron menores promedios de calidad de vida que aquellos sin trastorno mental.

## INTRODUCCIÓN

La discriminación percibida es aquella percepción que se tiene ante una actitud negativa o trato diferenciado debido a determinadas características particulares que poseen las personas según sexo, edad, condición social, entre otros, estando relacionada con resultados negativos sobre la salud ^1^. Asimismo, la calidad de vida es un constructo del bienestar psicológico, considerada equivalente al estado de salud e incluye componentes externos e individuales ^2^.

La relación entre discriminación percibida y calidad de vida ha sido estudiada en pacientes con enfermedades crónicas como el cáncer ^3^ o diabetes ^4^, y encontrando relaciones significativas cuando la discriminación percibida fue por raza o etnia ^3^. Así, en personas con algún trastorno mental, se ha encontrado relación significativa entre mayor estigma y peor calidad de vida ^5^.

En el Perú, la discapacidad que enfrentan las personas con trastornos mentales es importante ocupando el segundo lugar en el estudio de carga de enfermedad del 2019 ^6^, por lo que estudios sobre discriminación en lugares de la sierra se torna relevante por ser escasos y por enfocarse en personas con algún trastorno mental que hayan percibido discriminación por causas que no suelen ser estudiadas, y su relación con la calidad de vida ^7^.

En base a lo anterior, el objetivo fue determinar la relación entre la discriminación percibida y la calidad de vida en población adulta con algún trastorno mental, de Ayacucho, Cajamarca y Huaraz, tres ciudades de la sierra peruana.

**MENSAJE CLAVE**

Motivación para realizar el estudio. La discriminación como determinante social negativo para la salud, conlleva limitaciones en diversos ámbitos, incluso afectando la calidad de vida de las personas. Su investigación se desarrolla principalmente en el contexto racial. Estudios sobre otros tipos de discriminación son escasos en la literatura científica.

Principales hallazgos. Las personas con trastornos mentales percibieron discriminación por diversas condiciones sociodemográficas y presentaron menores puntajes de calidad de vida.

Implicancias. La discriminación como conducta excluyente se facilita y torna más compleja en personas con alguna vulnerabilidad.

## EL ESTUDIO

### Diseño de estudio

Estudio analítico transversal de base secundaria proveniente del Estudio Epidemiológico de Salud Mental de Ayacucho, Cajamarca y Huaraz del año 2017 del Instituto Nacional de Salud Mental «Honorio Delgado Hideyo Noguchi» (INSM «HD-HN») ^7^.

### Población y criterios de selección

El estudio primario estuvo conformado por adultos de 18 años a más de edad, residentes habituales en las ciudades de Ayacucho, Cajamarca y Huaraz. Se realizaron entrevistas directas e indirectas (ante algún problema en la comunicación). Se excluyeron a los que no desearon participar.

### Tamaño de muestra y muestreo.

El estudio primario consideró una población infinita, obteniéndose una muestra final de 3889 adultos ^7^. El muestreo fue probabilístico, complejo y trietápico (conglomerados de viviendas, viviendas y personas), siendo la unidad de análisis aquella persona que cumplía con los criterios de selección del estudio primario ^(^^7^. Cada ciudad del estudio constituyó un estrato, y en cada vivienda pudo seleccionarse a más de una persona de diferente unidad de análisis (adulto, adulto mayor, mujer unidad y adolescente) ^7^.

### Variables

*Persona con algún trastorno mental*

Esta variable fue elaborada a partir de otras variables que forman parte de la encuesta MINI (Encuesta Neuropsiquiátrica Internacional) versión en español 5.0.0, adaptada lingüísticamente para los estudios epidemiológico del INSM «HD-HN» ^7^. Los trastornos mentales considerados para el presente estudio se encuentran detallados en el material suplementario (S1). 

*Discriminación percibida*

Actitud de desvaloración hacia los otros, y que va desde el rechazo hasta conductas violentas, sin reconocer la igualdad de derechos entre las personas ^7^. Se mide a partir del Cuestionario de Discriminación ^8^. Esta variable fue incorporada en los cuestionarios epidemiológicos del INSM «HD-HN» a partir del estudio epidemiológico de salud mental en la sierra en el año 2003 ^9^, y analizado en su validez y confiabilidad (α de Cronbach=0,670) con la población de Iquitos, Pucallpa y Tarapoto ^10^. El cuestionario indaga por la prevalencia de vida y en los últimos 12 meses de sentirse rechazo o discriminado por diferentes características ^7^. Para el presente estudio solo se consideró la del último año: «En los últimos 12 meses, ¿con qué frecuencia ha sido Usted rechazado(a) o discriminado(a) por su sexo, edad, peso, color de piel, forma de vestir, condición económica o social, nivel educativo, religión, amistades, lugar de nacimiento, talla?, a partir de la cual se estimó la prevalencia en el último año». Esta última pregunta tuvo una respuesta en escala Likert (ninguna vez, rara vez, ocasionalmente, frecuentemente, muy frecuentemente) y que, para fines del presente estudio, se categorizó como variable dicotómica sí y no, correspondiendo a la categoría «no» aquellos que respondieron «ninguna vez». El contenido del cuestionario y adaptaciones se encuentran en el material suplementario (S3).

*Calidad de vida*

El índice de Calidad de Vida de Mezzich, elaborado en idioma español, fue adaptado y validado en Lima Metropolitana, y tiene una confiabilidad α de Cronbach de 0,870. Evalúa 10 áreas mediante una única pregunta con respuesta en escala Likert (que va desde 1=malo hasta 10=excelente) ^11^^,^^12^. El contenido del cuestionario y adaptaciones se encuentran en el material suplementario (S4).

*Características sociodemográficas de la población*

Se incluyó al sexo, edad, nivel de instrucción, religión y nivel de pobreza según autopercepción de cobertura de necesidades básicas con ingresos familiares ^7^. Las categorías consideradas y adaptaciones para el estudio se encuentran en el material suplementario (S2).

### Análisis estadístico

El procesamiento de datos se realizó utilizando el módulo de muestras complejas del programa SPSS V.21.0, este análisis se hizo con los datos ponderados. Se usó frecuencias y porcentajes para las variables cualitativas, y la mediana y rango intercuartílico para la calidad de vida. La comparación de las prevalencias de los distintos tipos de discriminación entre los dos subgrupos se realizó mediante las pruebas chi-cuadrado convertidas al estadístico F, y para comparar las dimensiones de calidad de vida y para evaluar las diferencias entre las medianas de la calidad de vida, según la discriminación en el último año, se aplicó la prueba U de Mann Whitney. El nivel de significancia (valor de p) se estableció en <0,05.

### Aspectos éticos

Al ser un estudio de base secundaria fue exonerado de revisión con Oficio N° 43-2023-CIEI/INSM «HD-HN» por el Comité Institucional de Ética en Investigación (CIEI) del INSM «HD-HN». Asimismo, el estudio primario, del que proviene la base de datos anonimizada, fue aprobado con oficio N°002-2016-CIEI/INSM «HD-HN» por el CIEI de la misma institución.

## HALLAZGOS

El total de participantes correspondió a 3889 adultos, de los cuales 1499 declararon tener algún trastorno mental. En la Tabla 1 se muestran las características sociodemográficas de los adultos de las ciudades de Ayacucho, Cajamarca y Huaraz.

**Tabla 1:** Características sociodemográficas de la población adulta de las ciudades de Ayacucho, Cajamarca y Huaraz, 2017.

Características de la población		Muestra total		Trastorno mental (n=1499)		Sin trastorno mental (n=2390)	
		n	%	n	%	n	%
Sexo							
	Hombre	1550	47,35	577	45,50	973	48,46
	Mujer	2339	52,65	922	54,50	1417	51,54
Grupo de edad							
	18 - 24	748	21,15	229	16,88	519	23,72
	25 - 44	1810	42,14	638	38,55	1172	44,30
	45 - 64	882	26,42	389	30,61	493	23,90
	65 a más	449	10,29	243	13,96	206	8,08
Grado de instrucción^a^							
	Sin instrucción/Inicial / Preescolar	201	5,06	110	7,05	91	3,86
	Primaria	606	14,78	259	17,37	347	13,23
	Secundaria/bachillerato	1171	29,13	457	29,43	714	28,94
	Superior no universitario	594	14,52	205	12,97	389	15,45
	Superior universitario/posgrado	1316	36,51	468	33,17	848	38,52
Religión							
	Católica	2866	73,45	1098	72,57	1768	73,98
	Evangélica	587	14,66	228	15,36	359	14,24
	Otras religiones	232	6,41	89	6,36	143	6,44
	Ninguna	204	5,48	84	5,72	120	5,33
Nivel de pobreza según autopercepción de cobertura de necesidades básicas con ingresos familiares^a^							
	Pobres extremos	55	1,21	27	1,21	28	1,20
	Pobres básicos	860	21,26	361	22,32	499	20,62
	No pobres básicos	2060	53,15	756	52,46	1304	53,57
	No pobres	904	24,38	353	24,01	551	24,61
Ciudades							
	Ayacucho	1188	22,70	567	45,28	728	36,39
	Cajamarca	1295	39,73	456	31,17	950	41,42
	Huaraz	1406	37,57	476	23,55	712	22,18

a Presentan valores perdidos (información ausente o faltante)

### Discriminación percibida según tipo de discriminación

La proporción de discriminación en el último año en el grupo de personas que manifestaron algún trastorno mental en comparación con los que no lo manifestaron resultó significativa, con excepción a la discriminación por la religión (p=0,802) y amistades (p=0,151) (Tabla 2).

**Tabla 2:** Prevalencia de discriminación percibida anual según tipos de discriminación en la población adulta que declaró tener algún trastorno mental de las ciudades de Ayacucho, Cajamarca y Huaraz, 2017.

Percepción de Discriminación anual según tipos de discriminación		Muestra total		Trastorno mental		Sin trastorno mental		Valor de p^a^
		n=3889	%	n=1499	%	n=2390	%	
Sexo								
	Sí	79	2,0	42	2,8	37	1,6	0,028
	No	3809	98,0	1457	97,2	2352	98,4	
Edad								
	Sí	127	3,5	75	5,2	52	2,5	0,001
	No	3762	96,5	1424	94,8	2338	97,5	
Peso								
	Sí	118	3,5	68	5,2	50	2,5	0,001
	No	3768	96,5	1431	94,8	2337	97,5	
Color de piel								
	Sí	42	1,1	24	1,7	18	0,7	0,015
	No	3847	98,9	1475	98,3	2372	99,3	
Forma de vestir								
	Sí	95	3,0	52	4,3	43	2,1	0,007
	No	3792	97,0	1447	95,7	2345	97,9	
Condición económica o social								
	Sí	143	3,5	76	5,0	67	2,7	0,001
	No	3743	96,5	1423	95,0	2320	97,3	
Nivel educativo								
	Sí	113	2,9	66	4,5	47	1,9	<0,001
	No	3764	97,1	1426	95,5	2338	98,1	
Religión								
	Sí	59	1,8	25	1,9	34	1,7	0,802
	No	3813	98,2	1465	98,1	2348	98,3	
Amistades								
	Sí	42	1,6	22	2,2	20	1,2	0,151
	No	3846	98,4	1476	97,8	2370	98,8	
Lugar de nacimiento								
	Sí	48	1,3	29	2,2	19	0,7	<0,001
	No	3840	98,7	1470	97,8	2370	99,3	
Talla								
	Sí	64	2,0	39	3,7	25	1,1	<0,001
	No	3824	98,0	1459	96,3	2365	98,9	

a Contraste de independencia para muestras complejas (pruebas chi-cuadrado convertidas al estadístico F).

### Calidad de vida

La mediana de la calidad de vida de la población estudiada fue de 7, 9 (RIC: 7,1-8,5) (Tabla 3).

Los adultos con algún trastorno mental obtuvieron una mediana de 7,6 (RIC: 6,8-8,3), resultando menor en comparación con aquellos no presentaron algún trastorno mental 8,0 (RIC: 7,3-8,7), siendo esta diferencia significativa (p<0,01) (Tabla 3).

**Tabla 3:** Calidad de vida en población adulta, que declaró tener algún trastorno mental, de las ciudades de Ayacucho, Cajamarca y Huaraz, 2017.

Calidad de vida	Muestra total			Trastorno mental			Sin trastorno mental			Valor de p^a^
	Mediana	Q1	Q3	Mediana	Q1	Q3	Mediana	Q1	Q3	
Promedio de calidad de vida total	7,9	7,1	8,5	7,6	6,8	8,3	8,0	7,3	8,7	<0,001
Bienestar físico	8,0	6,0	9,0	7,0	6,0	8,0	8,0	7,0	9,0	<0,001
Bienestar psicológico emocional	8,0	7,0	9,0	8,0	6,0	8,0	8,0	7,0	9,0	<0,001
Autocuidado y funcionamiento independiente	8,0	8,0	9,0	8,0	7,0	9,0	9,0	8,0	10,0	<0,001
Funcionamiento ocupacional	8,0	8,0	9,0	8,0	7,0	9,0	8,0	8,0	9,0	<0,001
Funcionamiento interpersonal	8,0	7,0	9,0	8,0	7,0	9,0	8,0	7,0	9,0	<0,001
Apoyo social emocional	8,0	7,0	9,0	8,0	6,0	8,0	8,0	7,0	9,0	<0,001
Apoyo comunitario y de servicios	7,0	5,0	8,0	6,0	5,0	8,0	7,0	6,0	8,0	<0,001
Plenitud personal	8,0	7,0	9,0	8,0	6,0	8,0	8,0	7,0	9,0	<0,001
Satisfacción espiritual	8,0	7,0	9,0	8,0	7,0	9,0	8,0	8,0	9,0	<0,001
Calidad de vida global	8,0	7,0	9,0	8,0	7,0	9,0	8,0	8,0	9,0	<0,001

a Prueba U de Mann-Whitney, Q1: cuartil 1, Q3: cuartil 3.

### Discriminación percibida y calidad de vida

Para la población con algún trastorno mental, el promedio de calidad de vida fue menor cuando se relacionó con la discriminación percibida en el último año en todos los tipos de discriminación (p<0,001) exceptuando el color de piel (p=0,326) y lugar de nacimiento (p=0,838) (Tabla 4). Hubo diferencia significativa en la calidad de vida promedio de las personas sin trastorno mental que percibieron discriminación en el último año, los promedios más bajos de calidad de vida se relacionaron con la discriminación percibida por peso (p<0,001), nivel educativo (p=0,003), forma de vestir (p=0,011), amistades (p<0,001), talla (p=0,013) y condición económica o social (p=0,034) (Tabla 4).

**Tabla 4:** Relación entre discriminación percibida anual y calidad de vida en población adulta en las ciudades de Ayacucho, Cajamarca y Huaraz, 2017.

Percepción de discriminación anual		Calidad de vida														
		Muestra total					Trastorno mental					Sin trastorno mental				
		n	Mediana	Q1	Q3	Valor de p^a^	n	Mediana	Q1	Q3	Valor de p^a^	n	Mediana	Q1	Q3	Valor de p^a^
Sexo																
	No	3763	7,90	7,20	8,50	<0,001	1444	7,60	6,80	8,30	<0,001	2316	8,00	7,40	8,70	0,050
	Sí	79	7,10	6,30	7,80		42	6,70	5,70	7,20		37	7,50	6,90	8,30	
Edad																
	No	3717	7,90	7,20	8,50	<0,001	1412	7,60	6,90	8,30	<0,001	2302	8,00	7,40	8,70	0,304
	Sí	126	7,30	6,40	8,10		74	6,70	5,80	7,90		52	7,80	6,80	8,50	
Peso																
	No	3722	7,90	7,20	8,60	<0,001	1418	7,60	6,80	8,30	<0,001	2301	8,00	7,40	8,70	<0,001
	Si	118	7,20	6,70	8,00		68	7,10	6,10	7,70		50	7,20	7,00	8,00	
Color de piel																
	No	3801	7,90	7,10	8,50	0.272	1462	7,60	6,80	8,30	0,326	2336	8,00	7,30	8,70	0,946
	Sí	42	7,50	6,70	8,40		24	6,90	6,70	8,00		18	8,20	6,80	8,40	
Forma de vestir																
	No	3746	7,90	7,20	8,50	<0,001	1434	7,60	6,80	8,30	<0,001	2309	8,00	7,40	8,70	0,011
	Sí	95	7,10	6,30	7,90		52	6,70	6,10	7,60		43	7,40	6,80	8,10	
Condición económica o social*																
	No	3697	7,90	7,20	8,50	<0,001	1411	7,60	6,80	8,30	0,001	2284	8,00	7,40	8,70	0,034
	Sí	143	7,30	6,40	8,20		75	7,00	6,40	7,90		67	7,90	7,10	8,40	
Nivel educativo																
	No	3718	7,90	7,20	8,50	<0,001	1413	7,60	6,80	8,30	<0,001	2302	8.00	7,40	8,70	0,003
	Sí	113	7,20	6,40	8,00		66	7,10	5,80	8,00		47	7,30	6,80	8,20	
Religión																
	No	3766	7,90	7,10	8,50	0,248	1452	7,60	6,80	8,30	0,008	2312	8,00	7,30	8,70	0,413
	Sí	59	7,60	7,00	8,60		25	6,80	5,70	7,60		34	8,00	7,40	8,90	
Amistades																
	No	3800	7,90	7,10	8,50	<0,001	1463	7,60	6,80	8,30	0,005	2334	8,00	7,40	8,70	<0,001
	Sí	42	6,90	6,00	7,40		22	6,30	5,70	7,30		20	7,40	6,90	7,50	
Lugar de nacimiento																
	No	3794	7,90	7,10	8,50	0,389	1457	7,60	6,80	8,30	0,838	2334	8,00	7,30	8,70	0,831
	Sí	48	7,90	6,70	8,50		29	7,50	6,40	8,50		19	8,00	6,80	8,70	
Talla																
	No	3778	7,90	7,20	8,50	<0,001	1446	7,60	6,80	8,30	0,008	2329	8,00	7,40	8,70	0,013
	Sí	64	7,10	6,30	7,70		39	7,00	5,80	7,50		25	7,40	7,00	8,00	

a Prueba U de Mann-Whitney.

## DISCUSIÓN

La discriminación en el último año en personas con algún trastorno mental fue mayor que en las personas sin trastorno mental, resultado parecido a una encuesta nacional del Perú, donde se encontró una mayor percepción de discriminación en personas con discapacidad intelectual o mental, entre otros ^13^. En el estudio de Alonso *et al.*^14^, en personas con trastornos mentales, encontraron que el 18% (n=1851) experimentó discriminación, incluso más que aquellas con problemas de salud física crónicas.

Los tipos de discriminación percibidos más frecuentemente reportados por adultos con algún trastorno mental fueron por condición socioeconómica o social, edad, peso y nivel educativo. Esto coincide con el estudio de Amirova *et al.*^15^, quienes hallaron en Inglaterra que lo más frecuente fue la discriminación por situación económica, y con una encuesta nacional chilena, donde la discriminación por situación socioeconómica (21%) y por edad (17%) fueron las más prevalentes ^16^. Amirova *et al.*^15^ también encontraron que, en Estados Unidos, la discriminación por sexo fue la más frecuente, principalmente en mujeres. Entre nuestros resultados destaca también la discriminación por sexo. Al respecto, debemos recordar que las mujeres en diversas partes del mundo se encuentran más expuestas al sexismo, que impacta de manera negativa en su calidad de vida ^17^, por lo que nuestros hallazgos podrían reflejar parte de la idiosincrasia cultural de las regiones de la sierra peruana.

Se encontraron diferencias significativas en la calidad de vida entre las dos submuestras, teniendo menores medianas los adultos con algún trastorno mental, principalmente en las dimensiones de la calidad de vida relacionada con el apoyo comunitario y el bienestar físico, estos resultados son compatibles con el estudio de Cedillo *et al*. ^18^, quienes encontraron que el incremento de las limitaciones en el funcionamiento de la persona con algún tipo de discapacidad disminuía su calidad de vida. Asimismo, en el estudio de Alonso *et al.*^14^, la menor calidad de vida en personas con trastornos mentales se asoció al estigma percibido.

Aunque se torna difícil realizar comparaciones por la escasez de estudios entre discriminación percibida y calidad de vida en población con algún trastorno mental, y por la utilización de diferentes escalas, se destaca el estudio de Ng *et al*. ^19^, en 380 personas adultas migrantes en Hong Kong, en quienes se encontró asociación negativa entre la discriminación percibida y tres dimensiones de la calidad de vida (salud física, psicológica y el medio ambiente) de las cuatro evaluadas con el cuestionario **«**WHOQOL-BREF**»**. Por otro lado, los diferentes tipos de discriminación interactúan entre sí, y que mediados por la experiencia discriminatoria, afectan la calidad de vida de las personas que los perciben ^17^. Aunque este enfoque de investigación no ha sido parte del nuestro se resalta para investigaciones futuras.

El presente estudio debe interpretarse considerando diversas limitaciones. Primero, al ser un estudio de fuente secundaria, la pregunta de investigación se respondió ciñéndose a los datos existentes. Otra limitación fue el sesgo de memoria (las preguntas recurren a los recuerdos del entrevistado). También el posible sesgo de medición (los instrumentos se validaron en castellano, aun cuando en la población del estudio existe quechua hablantes y con un contexto cultural distinto), y de deseabilidad social (por el carácter sensible de las preguntas). Cabe mencionar que en el estudio original se realizó una prueba piloto para obtener información válida y confiable ^7^. Pudo existir problema de comprensión sobre la discriminación por amistad, por un lado, puede hacer referencia a las amistades que tiene determinada persona, o que sus amistades la discriminan por diferentes motivos. Asimismo, al agruparse las respuestas en escala Likert de cinco a dos categorías, no se identificó si a mayor frecuencia de discriminación percibida había menor calidad de vida. Finalmente, no se pudo asociar la discriminación percibida y calidad de vida según a ciudad, debido a la ausencia de valores en alguno de los tres subgrupos formados.

Más allá de las limitaciones expuestas, el estudio tiene varias fortalezas, como contar con una muestra representativa, y ser uno de los primeros en comparar la relación entre discriminación percibida y la calidad de vida en personas con algún trastorno mental de ciudades de la sierra peruana, siendo meritorio el estudio por la escasa información sobre cuánto afecta la discriminación percibida en el bienestar.

En conclusión, las personas con trastornos mentales de las ciudades de Ayacucho, Cajamarca y Huaraz que percibieron discriminación en el último año presentaron menores promedios de calidad de vida que aquellos sin trastorno mental. Se recomienda continuar investigaciones sobre este determinante social que genera conflictos e influye negativamente en la consolidación del país.
